# Use of Sato's curved laryngoscope and an insulated-tip knife for endoscopic incisional therapy of esophageal web

**DOI:** 10.1111/den.12334

**Published:** 2014-09-17

**Authors:** Masahiko Ohtaka, Shouji Kobayashi, Takashi Yoshida, Tatsuya Yamaguchi, Tomoyosi Uetake, Tadashi Sato, Akira Hayashi, Mari Kanai, Takanori Yamamoto, Kyosuke Hatsushika, Keisuke Masuyama, Nobuyuki Enomoto

**Affiliations:** 1 First Department of Internal Medicine, University of YamanashiChuo, Japan; 2 Department of Otorhinolaryngology, University of YamanashiChuo, Japan

**Keywords:** endoscopic incision, esophageal web, insulated-tip knife (IT-knife), radial incision and cutting, Sato's curved laryngoscope

## Abstract

We experienced two cases of esophageal web accompanying severe stricture that were treated by endoscopic incisions with an insulated-tip knife (IT-knife). With attention paid to the mucosa at the stricture, the lesion was incised with an IT-knife without complications. Sato's curved laryngoscope was used even in cervical esophageal lesions and an excellent field was secured.

## Introduction

Endoscopic submucosal dissection (ESD) for gastric cancer has become widespread and this technique has also been applied to other treatment techniques, including endoscopic incisions for stricture after esophageal surgery^1^,^2^ and ESD.^3^ Here, we incised esophageal web-associated severe membranous stricture with an insulated-tip knife (IT-knife, KD-610-L; Olympus Medical Systems, Tokyo, Japan). With Sato's curved laryngoscope (curved laryngoscope; Nagashima Medical Instruments Co., Ltd, Tokyo, Japan), it was possible to secure a wide view from the hypopharynx to an esophageal inlet.^4^

## Case 1

**Figure 1:**
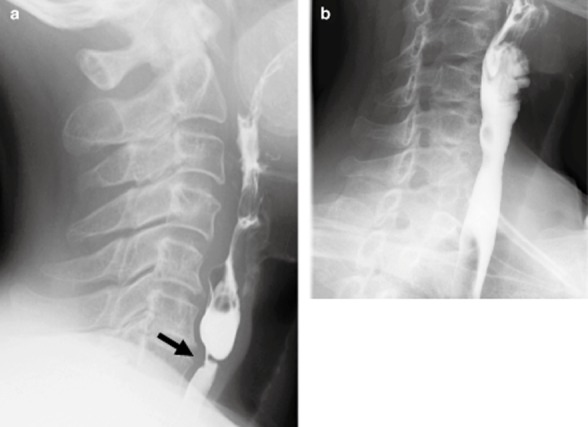
Esophagography of case 1 in lateral view. (a) Smooth circumferential stricture at the level of the lower edge of the sixth cervical vertebra before an endoscopic incision (arrow). (b) Marked alleviation of the stricture at 2 months after the endoscopic incision.

**Figure 2:**
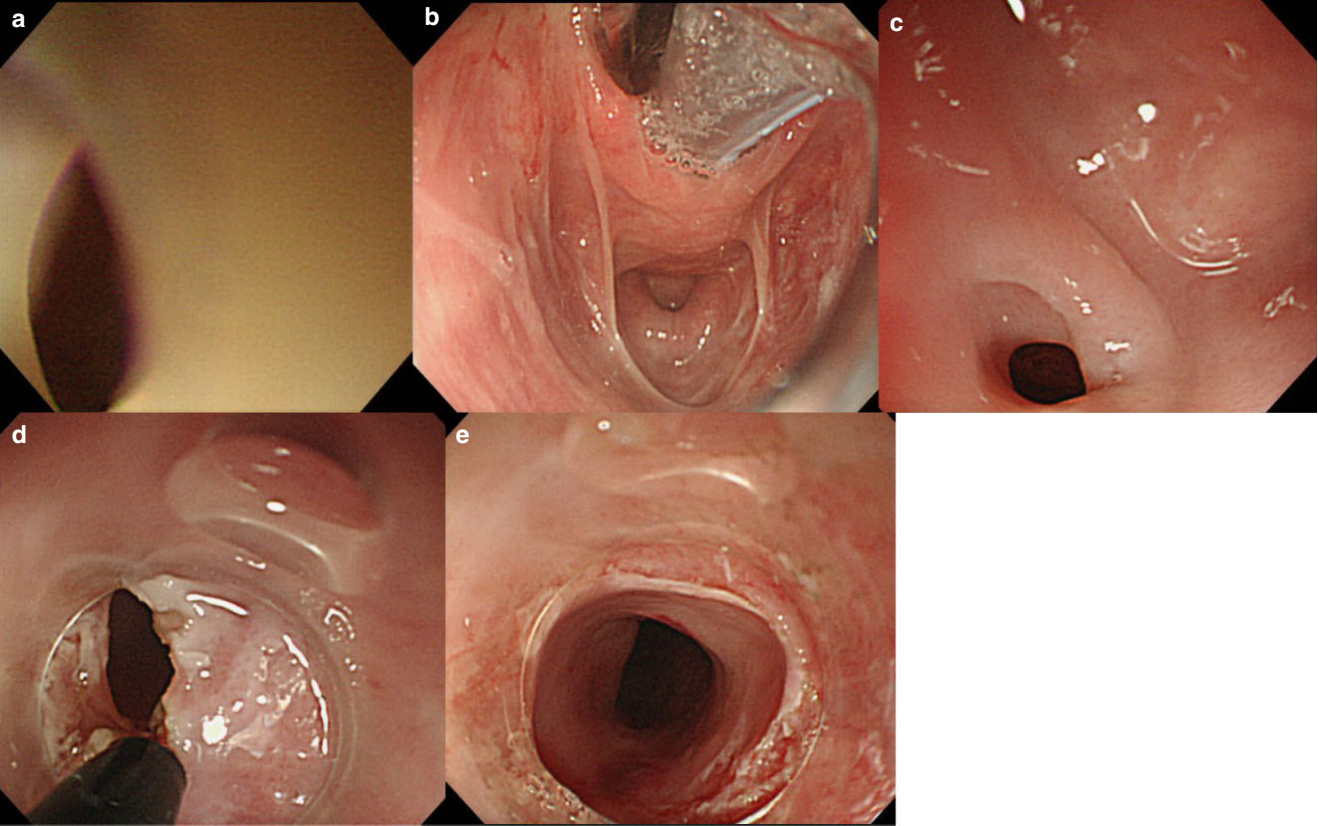
Esophagoscopy showed (a) membranous stricture at just below the left piriform recess. (b) Wide view in the hypopharynx using Sato's curved laryngoscope (Nagashima Medical Instruments Co., Ltd, Tokyo, Japan). (c) A membranous stricture appeared just at the distal side of the proximal esophageal ring. (d) IT-knife (Olympus Medical Systems, Tokyo, Japan) was used to radially incise the arch-shaped mucosa. (e) The esophageal membranous stricture improved.

A 58-year-old woman had gradually aggravating dysphagia for 5 years. She was able to take in small amounts of fluid but not solid meals. She was admitted to our hospital. She had no bodyweight loss. Her past history included uterine myoma and iron deficiency anemia when she was 40 years old. Physical examination showed no abnormal findings. Laboratory findings on admission showed a decreased number of red blood cells and an elevated iron level at 191 μg/dL, but total iron-binding capacity (TIBC) and ferritin were within normal limits. Esophagography in a lateral view showed a smooth circumferential stricture at the level of the lower edge of the sixth cervical vertebra (Fig. 1a). Esophagoscopy with XQ260NS (Olympus Medical Systems, Tokyo, Japan) revealed membranous stricture at just below the left piriform recess, and deeper insertion was unfeasible (Fig. 2a). Computed tomography (CT) showed non-specific thickening of the esophageal wall, but no tumor shadow. Taken together, an esophageal web was diagnosed. Endoscopic incisional therapy with an IT-knife was carried out.

A curved laryngoscope was inserted to secure a wide view in the hypopharynx under general anesthesia (Fig. 2b); the proximal esophageal ring was identified, and then the membranous stricture was located just at the distal side (Fig. 2c). A transparent attachment hood was applied to GIF-260J (Olympus), and the electrosurgical generator (Olympus ESG-100) was used with the Pulse Cut Slow mode at 20 W. An IT-knife was used to radially incise the arch-shaped mucosa (Fig. 2d). When the inner cavity of the distal side was observed, an incision became easier because the incision length could be confirmed. It was possible to pass the endoscope with a hood of 11.35 mm in diameter. The exposed submucosal layer had a circumferential length of approximately 3 mm (Fig. 2e). The procedure was completed without perforation or bleeding. To prevent re-stricture, a 14-Fr gastric tube was inserted. From postoperative days 4 and 7, the patient started drinking and taking fluid meals, respectively. On day 14, the gastric tube was removed and oral steroid, 20 mg/day, was started to prevent stricture; the dose was gradually decreased by 5 mg per week. No prophylactic balloon dilatation was done. Esophagography 2 months after the endoscopic incision showed marked alleviation of the stricture (Fig. 1b). She has taken meals without limitations for 22 months after surgery.

## Case 2

**Figure 3:**
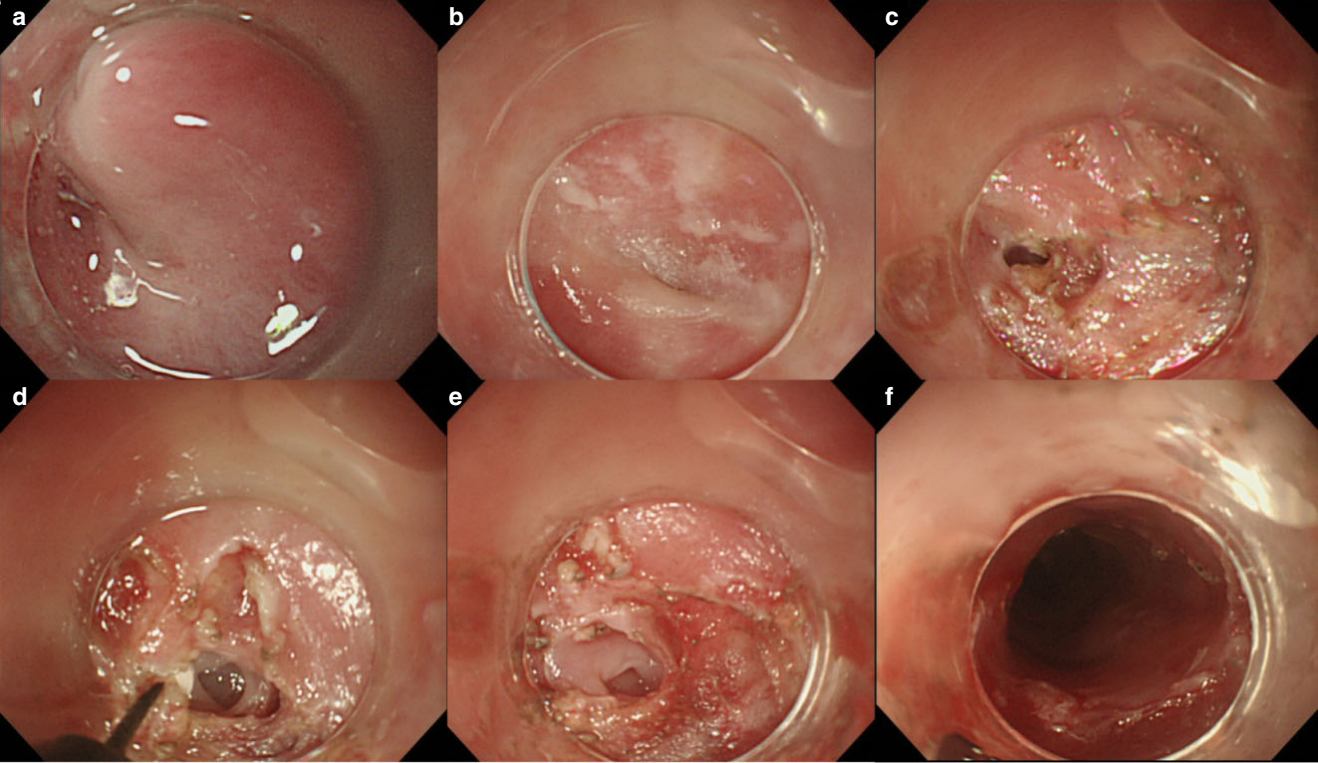
Esophagoscopy showed (a) no visible lumen at the piriform recess. (b) The post-cricoid part adhered to the posterior wall of the hypopharynx. (c) The IT-knife (Olympus Medical Systems, Tokyo, Japan) was applied to incise the pharyngeal adhesion. (d) An esophageal membranous stricture appeared. (e) An esophageal web was confirmed. (f) The esophageal web was incised and the esophageal membranous stricture improved.

**Figure 4:**
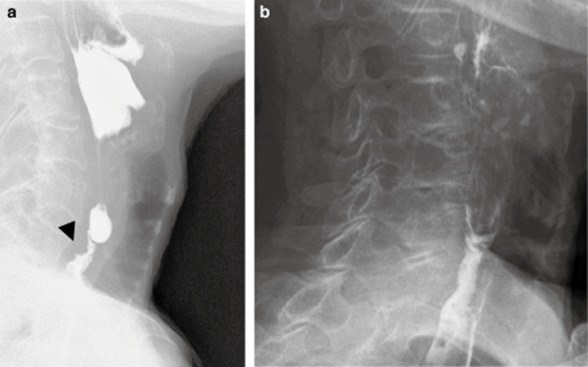
Esophagography of case 2 in lateral view. (a) Smooth circumferential stricture (arrow) at the level of the lower edge of the sixth cervical vertebra before an endoscopic incision. (b) Alleviation of the stricture at 12 months after the endoscopic incision.

A 76-year-old woman who had previously visited a clinic for a common cold when she was 50 years old was diagnosed with iron deficiency anemia at that time. She was unable to swallow tablets, so she received i.v. iron infusion. From the age of 75 years she had had dysphagia and had gradually become unable to eat. She was admitted to our hospital. No suicide attempt or corrosive esophagitis was recorded. Physical examination showed no abnormal findings. Laboratory findings on admission showed hypoalbuminemia and normocytic normochromic anemia. Esophagoscopy showed no visible lumen at the piriform recess and deeper insertion was unfeasible (Fig. 3a). Esophagography showed a smooth circumferential stricture at the level of the lower margin of the sixth cervical vertebra (Fig. 4a), and an esophageal web was diagnosed. When a hypopharyngeal view was secured with a curved laryngoscope, the post-cricoid part was seen to adhere to the posterior wall of the hypopharynx. At the pointed end of the piriform recess, it was impossible to insert the tip of the IT-knife (Fig. 3b). When the IT-knife was applied to the pharyngeal adhesion for an incision (Fig. 3c), an esophageal membranous stricture appeared (Fig. 3d), and an esophageal web was confirmed (Fig. 3e). Then, the arch-shaped mucosa of the esophageal web was radially incised (Fig. 3f), and the procedure was completed without perforation or bleeding. In order to prevent restenosis, an 18-Fr gastric tube was inserted. Hydrocortisone sodium succinate 300 mg was given for 3 days for mild laryngeal edema. From postoperative days 7 and 9, she started drinking and taking fluid meals, respectively. On day 9, the gastric tube was removed and oral steroid at 20 mg/day was started to prevent stricture; subsequently, the dose was gradually decreased by 5 mg per week. No prophylactic balloon dilatation was done. Esophagography 12 months after the endoscopic incision showed alleviation of the stricture (Fig. 4b). She has now taken meals without limitations for 18 months after surgery.

## Discussion

An esophageal web, a thin membranous tissue protruding into the esophageal lumen, pathohistologically comprises squamous epithelial cells and lamina propria mucosae. There are congenital webs (middle to lower parts) and acquired webs (cervical esophagus).^5^–^9^ Dysphagia, chest pain, and aspiration are the characteristic symptoms.^5^–^10^ For treatment, bougienage and balloon dilatation have generally been used.^6^–^9^ For bougienage, a guidewire is inserted through the stricture and a dilator is also passed under fluoroscopy.^6^ Alternatively, an endoscope with a small-caliber-tip transparent hood is passed through the web for bougienage.^7^ In balloon dilatation, a catheter with a balloon is inserted into the stricture under endoscopic guidance, and the balloon is inflated to dilate the stricture.^8^,^9^ Both techniques require from a single to a few sessions in order to increase the diameter between 15 mm and 20 mm. After dilatation by both techniques, the dilated area is carefully inspected to assess transmural esophageal injury.

Endoscopic electrosurgical incision of the esophageal web with a cutting snare was reported for the first time in congenital cases with thoracic stricture.^5^ There was only one case report in 2011 by Seo *et al*. that described an endoscopic incision of a cervical esophageal web with an IT-knife.^10^ A 47-year-old woman had complained of dysphagia for 20 years. The mucosal fold of a web adjacent to the proximal esophageal ring was incised in three directions with an IT-knife 2. No complication occurred and subjective symptoms improved later. The present cases were treated in the same manner. It is a characteristic property of the IT-knife that an insulated ceramic ball is attached at the tip of the electrosurgical needle knife. Therefore, although the anal side of the stricture is difficult to see, the mucosa is protected from injury by the insulated ball. To make the best use of this feature, an IT-knife was used instead of an IT-knife 2 with electrodes stretched radially in three directions. The blade, from the insulated part of the tip to the sheath, was applied to the mucosa at the stricture for an incision, and the stricture was successfully resolved.

An endoscopic incision with an IT-knife is a new technique for treatment of postoperative anastomotic stricture. Lee *et al*. reported in 2009 that 24 cases were treated by the technique and followed up for 24.08 months on average: 87.5% of the cases improved after one session, and the remaining cases developed no recurrence of stricture.^1^ In Japan, Muto *et al*. named the technique radial incision and cutting (RIC).^2^ They carried out RIC in 25 cases, and 56% could take solid meals 6 months after one session of RIC, and eventually 72% required no balloon dilatation. Two cases developed perforation (4% of 49 RIC sessions in total), but both cases were successfully treated by conservative therapy.

Balloon dilation bluntly and blindly expands the constricted mucosa. We rely on balloon size and expansion speed for adjustment in the extending range. However, there is not enough evidence to recommend that dilation should always be carried out by an endoscopic incision. In an endoscopic incision with good surgical view, we can incise while observing directly the incision site. Therefore, it is possible to reduce the risk for perforation because we can adjust the size of the incision. In our cases, the curved laryngoscope, which is often used in endoscopic laryngopharyngeal surgery (ELPS), was used to secure a field of vision. ELPS is a transoral technique with an endoscope that is one of the possible operations for superficial pharyngeal carcinoma.^4^ The tip of the curved laryngoscope is set just above the vocal cords, and the larynx is subsequently lifted forward. Thus, in most cases, we can ensure a wide field of view up to the esophageal inlet under general anesthesia.^4^ In particular, in case 1, carrying out balloon dilatation was not difficult and inserting the guidewire was also easy. However, we knew already that a good view in front of the esophageal inlet could be secured in this way. In case 2, as adhesion between the posterior wall of the hypopharynx and post-cricoid part was suspected, a curved laryngoscope was useful in securing a field of vision and evaluating the stricture site. A curved laryngoscope must be considered an appropriate tool to facilitate the treatment of a lesion at the cervical esophagus.
